# A call to safeguard sexual and reproductive health information and services during Ebola outbreaks

**DOI:** 10.1186/s13031-021-00392-3

**Published:** 2021-07-10

**Authors:** Nguyen Toan Tran, Désirée Lichtenstein, Benjamin Black, Alice Rosmini, Catrin Schulte-Hillen

**Affiliations:** 1 Australian Centre for Public and Population Health Research, Faculty of Health, University of Technology Sydney, Sydney, NSW Australia; 2 Faculty of Medicine, University of Geneva, Geneva, Switzerland; 3 United Nations Population Fund, Humanitarian Office, Geneva, Switzerland; 4 Médecins Sans Frontières, Doctors Without Borders (MSF), London, UK; 5 United Nations Population Fund, West and Central Africa Regional Office, Dakar, Senegal

## Abstract

The recent Ebola virus disease (EVD) outbreaks in 2021 exemplify how sexual and reproductive health services are too often considered unessential during health emergencies. Bleeding for reasons other than EVD, such as pregnancy complications or rape, can be construed as EVD symptoms, reinforcing fear and stigmatisation, and delaying timely access to adequate care. In this commentary, we urgently call on all humanitarian actors to integrate the Minimum Initial Services Package for Sexual and Reproductive Health in Crisis Situations into current and future EVD preparedness and response efforts.

## Background

Sexual and reproductive health (SRH) conditions are among the leading causes of mortality in women of reproductive age, with countries beset by fragility and low-resilience health systems accounting for two-thirds of maternal deaths worldwide [1]. Ebola virus disease (EVD) is a severe zoonotic illness with an average case fatality rate of 50%. It spreads among humans through bodily fluids. Past EVD outbreaks suggest that halting health-care services judged to be unrelated to the epidemic response caused more deaths than did the epidemic itself [2].

At the intersection of EVD and SRH, poor health outcomes arise from the disruption of life-saving services, including maternal and neonatal care and care for individuals experiencing abortion complications, sexual violence, and other forms of gender-based violence [3]. Women and girls are at risk of gender-based violence due to increased exposure to perpetrators during curfews, school closure, and economic hardship, among others [4]. The mortality among EVD-positive pregnant women is not higher than that of non-pregnant individuals with EVD [5]. However, almost all EVD-affected pregnancies end in miscarriage, stillbirths, or neonatal deaths [6]. EVD-positive pregnant women can show signs and symptoms not meeting EVD case definitions [7]. Conversely, bleeding for reasons other than EVD, such as pregnancy, rape, and menses, can be misinterpreted as EVD symptoms, reinforcing fear and stigmatisation by the community and health-care providers, delaying timely access to adequate care [8].

The 2021 EVD outbreaks in Guinea and the Democratic Republic of Congo exemplify how regional and national humanitarian actors must actively safeguard SRH interventions in contingency plans, resource mobilisation, and responses. Consequently, the Inter-Agency Working Group for Reproductive Health in Crises issued field guidance on SRH and EVD [9]. The guidance builds on the prerequisite that health ministries and humanitarian stakeholders, including SRH leaders, should coordinate and plan to ensure that EVD preparedness and response integrate SRH services, supplies, and referral mechanisms. To enable the systematic prioritisation of SRH in current and future EVD responses—and minimise SRH-related deaths—the following recommendations should also be considered.

First, all essential services and supplies defined by the Minimum Initial Services Package (MISP) for SRH in Crisis Situation, a standard in humanitarian response, should be prioritised during the whole EVD response [10]. It includes intrapartum care, emergency obstetric and neonatal care, post-abortion care, safe abortion care to the full extent of the law, contraception, care for rape survivors, and prevention and treatment of HIV and other sexually-transmitted infections. Women and adolescent girls with suspected EVD should be offered pregnancy testing at EVD triage posts—backed by standard operating procedures that guarantee safe and dignified isolation until EVD ascertainment and effective referrals of confirmed cases to EVD treatment centres [11]. These centres should have adequate SRH supplies, personnel, and protocols, including for emergency obstetric and neonatal care, and offer counselling on potential outcomes and options to individuals testing positive for EVD and pregnancy. Equally important is safer sex and contraceptive counselling for all individuals of reproductive age, regardless of EVD and pregnancy status.

Second, engaging health-care workers, EVD survivors, and representatives of the community and targeted audiences is crucial for crafting clear and relevant public health information. This information should reiterate the importance of seeking care for childbirth and other SRH needs and medical emergencies. Risk communication and community engagement strategies should cover SRH and EVD, the risk of poor health outcomes for women and girls, help individuals differentiate “inexplicable bleeding” (EVD) from “explicable bleeding” (e.g., menses)—and address related stigma. No less critical is information about contraception, safer sex, breastfeeding, avoiding unsafe home births, and where to access care, including for Ebola virus vaccine.

Third, infection prevention and control measures, including personal protective equipment, should be part of managing labour and deliveries, abortion-related care, and any procedures with bodily fluid contact, such as rape survivor care, regardless of EVD status. Reliable supplies to enforce such precautions in both EVD treatment centres and SRH facilities should be thoroughly planned during coordination meetings. These facilities should also optimise waste management practices of used menstrual hygiene products and pregnancy-related fluids and tissues, including safe placenta disposal. Not least, SRH providers, including community midwives and other frontline health-care workers, must be prioritized in national Ebola vaccination programmes.

## Conclusion

Our guidance on SRH and EVD was issued to galvanise country and regional stakeholders to safeguard essential health-care services, including SRH, during EVD preparedness and response efforts. We call upon them to treat life-saving SRH services as core constituents of the overall EVD intervention package, one that does not enfeeble but bolsters health-care systems while meeting the health needs of women, girls, and the whole community.

## Data Availability

Not applicable.

## Abbreviations

EBD: Ebola virus disease
SRH: Sexual and reproductive health
